# Genome-informed loop-mediated isothermal amplification assay for specific detection of *Pectobacterium parmentieri* in infected potato tissues and soil

**DOI:** 10.1038/s41598-021-01196-4

**Published:** 2021-11-09

**Authors:** Ryan Domingo, Cristian Perez, Diksha Klair, Huong Vu, Alika Candelario-Tochiki, Xupeng Wang, Amihan Camson, Jaclyn Nicole Uy, Mouauia Salameh, Dario Arizala, Shefali Dobhal, Gamze Boluk, Jon-Paul Bingham, Francisco Ochoa-Corona, Md Emran Ali, James P. Stack, Jacqueline Fletcher, Jenee Odani, Daniel Jenkins, Anne M. Alvarez, Mohammad Arif

**Affiliations:** 1 Department of Plant and Environmental Protection Sciences, University of Hawaii at Manoa, Honolulu, HI USA; 2 Department of Tropical Plant and Soil Sciences, University of Hawaii at Manoa, Honolulu, HI USA; 3 Department of Chemistry, University of Hawaii at Manoa, Honolulu, HI USA; 4 Department of Molecular Biosciences and Bioengineering, University of Hawaii at Manoa, Honolulu, HI USA; 5 Department of Entomology and Plant Pathology, Oklahoma State University, Stillwater, OK USA; 6 Department of Plant Pathology, University of Georgia, Tifton, GA USA; 7 Department of Plant Pathology, Kansas State University, Manhattan, KS USA; 8 Department of Human Nutrition, Food and Animal Sciences, University of Hawaii at Manoa, Honolulu, HI USA

**Keywords:** Bacteria, Bacteriology, Pathogens

## Abstract

*Pectobacterium parmentieri* (formerly *Pectobacterium wasabiae*), which causes soft rot disease in potatoes, is a newly established species of pectinolytic bacteria within the family *Pectobacteriaceae*. Despite serious damage caused to the potato industry worldwide, no field-deployable diagnostic tests are available to detect the pathogen in plant samples. In this study, we aimed to develop a reliable, rapid, field-deployable loop-mediated isothermal amplification (LAMP) assay for the specific detection of *P. parmentieri*. Specific LAMP primers targeting the *petF1* gene region, found in *P. parmentieri* but no other *Pectobacterium* spp., were designed and validated in silico and in vitro using extensive inclusivity (15 strains of *P. parmentieri*) and exclusivity (94 strains including all other species in the genus *Pectobacterium *and host DNA) panels. No false positives or negatives were detected when the assay was tested directly with bacterial colonies, and with infected plant and soil samples. Sensitivity (analytical) assays using serially diluted bacterial cell lysate and purified genomic DNA established the detection limit at 10 CFU/mL and 100 fg (18–20 genome copies), respectively, even in the presence of host crude DNA. Consistent results obtained by multiple users/operators and field tests suggest the assay’s applicability to routine diagnostics, seed certification programs, biosecurity, and epidemiological studies.

## Introduction

Potato blackleg and soft rot, caused by bacterial species in the genera *Pectobacterium* and *Dickeya*, are among the most significant diseases with large economic impacts on potato crop production^1–4^. Soft rot causes one billion dollars’ loss to the potato industry annually^5^. *Pectobacterium* comprises eighteen species that utilize pectinolytic and cellulolytic enzymes to infect a broad range of crop and non-crop plants under wet and semi-anaerobic conditions^6^. *Pectobacterium parmentieri*, previously known as *P. wasabiae,* a gram-negative, rod-shaped virulent pectolytic pathogenic bacterium^7^, commonly isolated from potato plants and tubers displaying blackleg and soft rot symptoms, is able to survive under a range of environmental conditions. Latently infected potato seed-tuber and contaminated propagative plant materials contribute to the dispersal of *P. parmentieri;* pathogen has been identified in several regions of Europe^8^, Canada^9^, United States^10^, New Zealand, China^11^ and South Africa^12^.

Since soft rot diseases are caused by a complex of bacterial pathogens of different species and phenotypes, detection of an individual bacterial species requires a robust, accurate diagnostic tool^13^. At present, there are no validated methods reported for the specific detection of *P. parmentieri.* PCR-based methods are sensitive and specific, however, they can be time-consuming and are confined to laboratory settings with specialized equipment^14^. There are isothermal methods that can be used in field conditions and have advantages over the PCR-based methods, for example, recombinase polymerase amplification (RPA)—less sensitive to inhibitors and eliminates the need for DNA isolation^15,16^. Previously RPA was used to differentiate between *Pectobacterium* and *Dickeya,* but did not specifically identify the species *P. parmentieri*^17^. The cost per RPA reaction is higher than for other field-deployable techniques, such as loop-mediated isothermal amplification (LAMP). The LAMP assay has gained popularity for pathogen detection and point-of-need application^18,19^. This isothermal nucleic acid amplification technique is based on auto-cyclic amplification and a high DNA strand displacement activity facilitated by a *Bacillus stearothermophilus (Bst)* polymerase^18,20^. LAMP is typically performed at 65 °C, a temperature ideal for *Bst* polymerase activity^21^. LAMP provide a sensitive and straightforward detection suitable for field applications that doesn’t require expensive reagents or sophisticated equipment. Pathogen detection by LAMP can be achieved in 10 to 20 min, and the amplified products can be observed visually with SYBR Green dye^22,23^.

Several closely related *Pectobacterium* species cause soft rot and blackleg diseases in potatoes and shared high pairwise homology in their genomic regions. Therefore, it is crucial to identify the signature genomic region for designing taxon-specific primers^24–27^. The comparative genomic analysis allows identification of unique and conserved genomic regions suitable for a robust and highly specific diagnostic assay^22,26^. Primer specificity for *P. parmentieri* is important since it occurs in highly heterogeneous populations in different geographic locations^28^. This selectivity eliminates cross-reactivity with non-target pathogens..

This study described the development of a LAMP assay for specific detection of *P. parmentieri* in infected potato tissues and soil samples. Both field and laboratory assays confirmed the robustness of the diagnostic method. The resulting protocol is simple for field applications, routine diagnostics, surveillance, biosecurity, epidemiology, and disease management to mitigate the damaging effects and economic losses caused by *P. parmentieri* in agricultural production.

## Results

### Target selection and primer in-silico specificity

**Figure 1:**
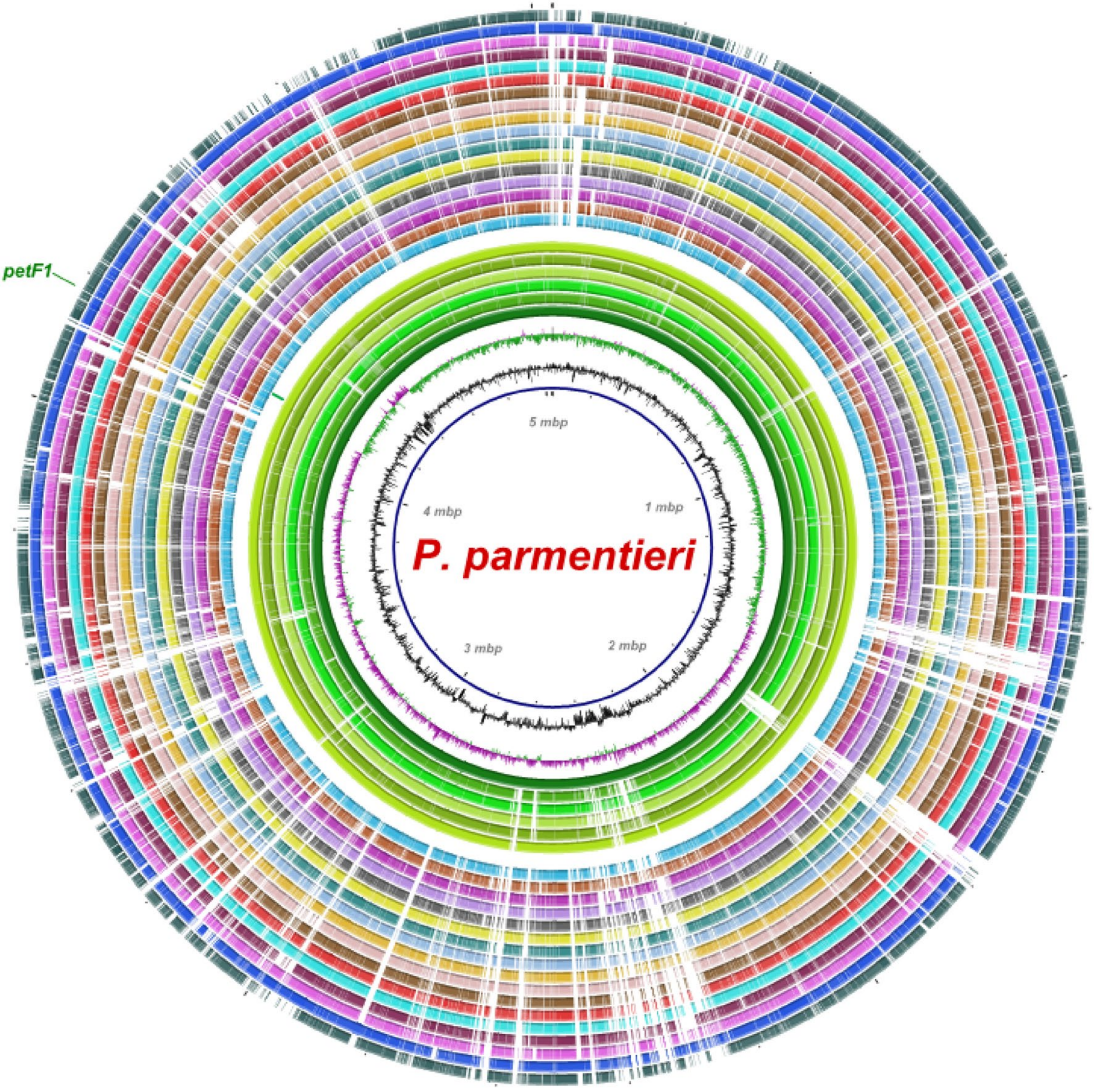
Locus representation of the target gene *petF1* (ferredoxin) used for the specific detection of *Pectobacterium parmentieri*. The BLAST ring image was generated using the BRIG software^29^. The circular graphic shows the multiple alignment and genome comparison of six *P. parmentieri* strains and the other seventeen species that currently encompass the *Pectobacterium* genus. The three innermost layers in the graphic portray the genome coordinates (mega base pairs—*mbp*), GC content (zigzag black line) and GC skew (purple + /green − zigzag) of the *P. parmentieri* RNS 08-42-1A reference genome. The other colored rings, from the innermost to outermost, depict the nucleotide BLAST alignment of *P. parmentieri* RNS 08-42-1A (NZ_CP015749), *P. parmentieri* SCC3193 (NC_017845), *P. parmentieri* WPP163 (NC_013421), *P. parmentieri* IFB5619 (NZ_CP026985), *P. parmentieri* HC (NZ_CP046376), *P. parmentieri* IFB5486 (NZ_CP026982), locus of *petF1* uniquely present in *P. parmentieri* (green line and label), *P*. *actinidiae* KKH3 (NZ_JRMH00000000), *P. aquaticum* A212-S19-A16 (NZ_QHJR00000000), *P. aroidearum* PC1 (NC_012917), *P. atrosepticum* JG10-08 (NZ_CP007744), *P. betavasculorum* NCPPB 2795 (NZ_JQHM00000000), *P. brasiliense* SX309 (NZ_CP020350), *P. carotovorum* WPP14 (NZ_CP051652), *P. fontis* M022^T^ (JSXC00000000), *P. odoriferum* BC S7 (NZ_CP009678), *P. parvum* s0241 (OANP00000000), *P. peruviense* IFB5232 (NZ_LXFV00000000), *P. polaris* NIBIO 1006 (NZ_CP017481), *P. polonicum* DPMP315 (NZ_RJTN00000000), *P. punjabense* SS95 (NZ_CP038498), *P. versatile* 3–2 (NZ_CP024842), *P. wasabiae* CFBP 3304 (NZ_CP015750) and *P. zantedeschiae* 2 M (NZ_PESL00000000).

A signature region of the ferredoxin gene *petF1*, was selected to design and develop a highly specific and robust assay. The signature region within *petF1* was not found in any other species of *Pectobacterium* but it was detected within all genomes of *P. parmentieri*. Six LAMP primers designed using *petF1* gene matched 100% in query coverage and identity with all *P. parmentieri* genomes available publicly in the NCBI GenBank genome sequence database (Table 1). No crossmatch was observed with any other sequence present in the database. The *petF1* gene region in *P. parmentieri,* is indicated in the BLAST Ring Image Generator (BRIG) image (Fig. 1), which includes genomes from *P. parmentieri* and other species of *Pectobacterium*.

**Table 1:** Primers designed using *petF1* gene region to develop loop-mediated isothermal amplification assay for specific and rapid detection of *Pectobacterium parmentieri*.

Primer name	Sequence (5ʹ–3ʹ)	Length (nt)	GC (%)
PP-F3	ATCATCGATGCTGCAGAA	18	44
PP-B3	ACATCAGAGGTTGGATATGC	20	45
PP-FIP	AGACACACGCAAGTAGAGCAAGCAGGTGTTGAACTTCC	38	50
PP-BIP	TGCGATCTCGGGAACTTATGATATACATGCCAAAAGGTATCCTT	44	41
PP-LF	CTCCAGCCCTACAACTATA	19	47
PP-LB	TAGATGATGAGCAAATTAGT	20	30

### Specificity of LAMP assay

**Figure 2:**
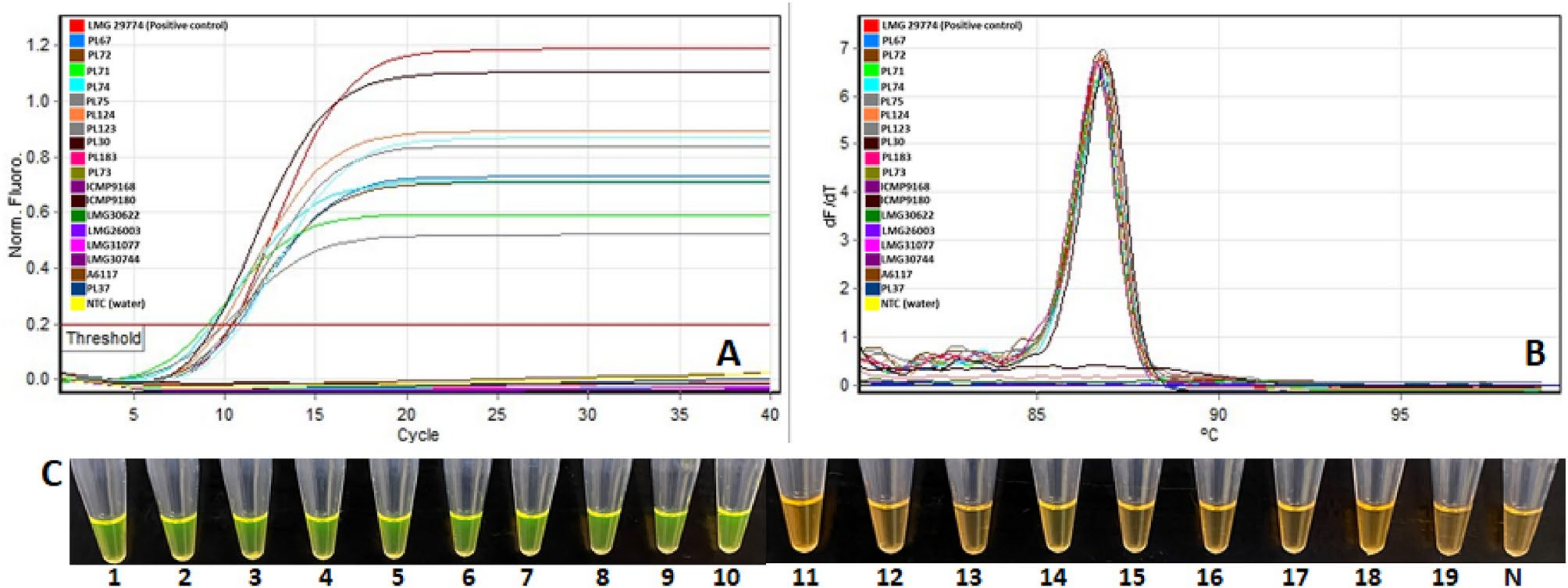
Specificity assay of loop-mediated isothermal amplification (LAMP) for specific detection of *Pectobacterium parmentieri*. Ten representative strains of *P. parmentieri* and 10 representative strains from the exclusivity panels are shown. (**A**) Real-time amplification plot with strains from both inclusivity and exclusivity panels; (**B**) melt-curve of 10 strains of *P. parmentieri*, no melt curve was observed with the strains from exclusivity panel and negative controls; (**C**) visualization of LAMP products after adding 3 μL of SYBR Green I stain. Tube 1, positive control *P. parmentieri* (LMG29774), tubes 2–10 *P. parmentieri* (PL67, PL72, PL71, PL74, PL75, PL124, PL123, PL30, and PL183), tubes 11–19 *P. carotovorum* (PL 73), *P. versatile* (ICMP 9168), *P. polaris* (ICMP 9180), *P. punjabense* (LMG 30,622), *P. actinidae* (LMG 26,003), *P. polonicum* (LMG31077), *P. fontis* (LMG30744), *Ralstonia solanacearum* (A6117), *Xanthomonas phaseoli* pv. *dieffenbachiae* (PL37), and N, negative template control (NTC, water).

The effectiveness of the LAMP assay was demonstrated with extensive inclusivity and exclusivity panels (Tables 2, 3). The LAMP assay detected all 15 strains of *P. parmentieri* (mostly from North America) represented in the inclusivity panel (Fig. 2, Table 2). The exclusivity panel consisted of 94 bacteria, including strains from different but closely related genera, a complete representation of all other described species of *Pectobacterium*, and healthy potato DNA (Table 3). Sigmoid-shaped curves (Fig. 2A) and bell-shaped melt curves (Fig. 2B) were observed. Reaction tubes containing LAMP products from *P. parmentieri* strains changed color from orange to green after the addition of 3 ul of SYBR Green I (Fig. 2C). Ten strains of *P. parmentieri,* which included LMG29774, PL67, PL72, PL71, PL74, PL75, PL124, PL123, PL30, and PL183, were used to represent the inclusivity panel. The samples representing the exclusivity panel (Fig. 2) included *P. carotovorum* (PL 73), *P. versatile* (ICMP 9168), *P. polaris* (ICMP 9180), *P. punjabense* (LMG 30,622), *P. actinidae* (LMG 26,003), *P. polonicum* (LMG31077), *P. fontis* (LMG30744), *Ralstonia solanacearum* (A6117), *Xanthomonas phaseoli* pv. *dieffenbachiae* (PL37), and negative template control (NTC, water). No amplification was observed from bacterial strains of other *Pectobacterium* sp., *Dickeya* sp., other gram-positive bacteria, endophytes/saprophytes isolated from potato or healthy potato plants (Fig. 2, Table 2). These data indicate that no cross-reactivity occurred with non-target bacterial species.

**Table 2:** Bacterial strains included in the inclusivity panel for validation of the loop-mediated isothermal amplification assay developed for specific and rapid detection of *Pectobacterium parmentieri*.

Species	Strain ID	Other associated name	Host/Source	Origin	LAMP results
*Pectobacterium parmentieri*	LMG29774	–	*Solanum tuberosum*	France	+
*P. parmentieri*	PL74	PS59A	*S. tuberosum*	Hawaii, USA	+
*P. parmentieri*	PL67	PS22B	*S. tuberosum*	Hawaii, USA	+
*P. parmentieri*	PL72	PS47B	*S. tuberosum*	Hawaii, USA	+
*P. parmentieri*	PL71	PS42	*S. tuberosum*	Hawaii, USA	+
*P. parmentieri*	PL75	PS63A	*S. tuberosum*	Hawaii, USA	+
*P. parmentieri*	PL124	PS38D	*S. tuberosum*	Hawaii, USA	+
*P. parmentieri*	PL123	PS38A	*S. tuberosum*	Hawaii, USA	+
*P. parmentieri*	PL30	GBp2-1	*S. tuberosum*	Hawaii, USA	+
*P. parmentieri*	PL183	W1-98–2	*S. tuberosum*	Hawaii, USA	+
*P. parmentieri*	A1852	M784	*S. tuberosum*	Colorado, USA	+
*P. parmentieri*	PL70	PS38F	*S. tuberosum*	Hawaii, USA	+
*P. parmentieri*	WPP168	A6159	*S. tuberosum*	Wisconsin, USA	+
*P. parmentieri*	WPP163	–	*S. tuberosum*	Wisconsin, USA	+
*P. parmentieri*	PL128	13B	*S. tuberosum*	Hawaii, USA	+

Plus (+) sign indicates positive LAMP amplification; – indicates that information is not available.

**Table 3:** Bacterial strains and healthy plant host samples used in the exclusivity panel for validation of loop-mediated isothermal amplification assay developed for specific and rapid detection of *Pectobacterium parmentieri*.

Species	Strain ID	Other associated name	Host/source	Origin	LAMP results
**Closely related species**					
*Pectobacterium cypripedii*	LMG 1268	–	*Cypripedium* sp.	USA	Negative
*P. aroidearum*	LMG 2417	–	*Zantedeschia aethiopica*	South Africa	Negative
*P. betavasculorum*	LMG 2461	–	*Beta vulgaris*	USA	Negative
*P. betavasculorum*	LMG 2466	–	*B. vulgaris*	USA	Negative
*P. betavasculorum*	A3000	–	–	–	Negative
*P. peruviense*	LMG 30,269	A6300	*S. tuberosum*	Peru	Negative
*P. atrosepticum*	LMG 2386	A6324	*S. tuberosum*	United Kingdom	Negative
*P. atrosepticum*	LMG 2375	A6280	*S. tuberosum*	United Kingdom	Negative
*P. atrosepticum*	A2998	–	–	–	Negative
*P. cacticida*	LMG 17,936	A6334	*Carnegiea gigante*a	USA	Negative
*P. punjabense*	LMG 30,622	A6339	*S. tuberosum*	Pakistan	Negative
*P. actinidiae*	LMG 26,003	A6337	*Actinidia chinensis*	Korea	Negative
*P. polonicum*	LMG 31,077	A6343	Ground water from potato field	Poland	Negative
*P. fontis*	LMG 30,744	A6340	Fresh water	Malaysia	Negative
*P. zantedeschiae*	CFBP 1357	A6316	*Zantedeschia* sp.	France	Negative
*P. parvum*	CFBP 8631	A6318	*S. tuberosum*	Finland	Negative
*P. polaris*	ICMP 9180	A6344	*S. tuberosum*	Netherlands	Negative
*P. aquaticum*	CFBP 8637	A6319	Environment/fresh water	France	Negative
*P. versatile*	ICMP 9168	A6345	*S. tuberosum*	Netherlands	Negative
*P. wasabiae*	PL188	WI_127_2p	*S. tuberosum*	Hawaii, USA	Negative
*P. wasabiae*	PL190	WI_380	*S. tuberosum*	Hawaii, USA	Negative
*P. wasabiae*	Wis_A1438	CFBP 3304	*Eutrema wasabi*	Japan	Negative
*P. brasiliense*	PL63	K-G	*Brassica oleracea* var. *sabellica*	Hawaii, USA	Negative
*P. brasiliense*	PL184	WI_367_1	*S. tuberosum*	Hawaii, USA	Negative
*P. brasiliense*	A6149	WPP5	*S. tuberosum*	Wisconsin, USA	Negative
*P. odoriferum*	A1089	QR-11	*Capsicum* sp.	California, USA	Negative
*P. odoriferum*	A2686	E43	*B. oleraceae* var. *capitata*	Hawaii, USA	Negative
*P. carotovorum*	PL73	PS51C	*S. tuberosum*	Hawaii, USA	Negative
*P. carotovorum*	PL185	WI_99_2	*S. tuberosum*	Hawaii, USA	Negative
*P. carotovorum*	PL186	WI_98_1	*S. tuberosum*	Hawaii, USA	Negative
*P. carotovorum*	PL187	WI_451_2	*S. tuberosum*	Hawaii, USA	Negative
*P. carotovorum*	PL182	WI_127_1a	*S. tuberosum*	Hawaii, USA	Negative
*P. carotovorum*	PL189	WI-539	*S. tuberosum*	Hawaii, USA	Negative
*P. carotovorum*	A5280	1-#31	Irrigation water	Hawaii, US	Negative
*P. carotovorum*	A5278	1-#21	Irrigation water	Hawaii, US	Negative
*Pectobacterium* sp*.*	PL34	–	*Hoodia* sp.	Hawaii, USA	Negative
*P. versatile*	PL62	–	*S. tuberosum*	Hawaii, USA	Negative
*P. versatile*	A1838	UC 202.1B	*S. tuberosum*	California, USA	Negative
*Dickeya aquatica*	LMG 27,354	A6293	River water	United Kingdom	Negative
*D. solani*	LMG27549	A6294	*S. tuberosum*	Ireland	Negative
*D. solani*	LMG27552	A6296	*S. tuberosum*	United Kingdom	Negative
*D. fangzhongdai*	CFBP 8607	A6317	*Pyrus communis*	China	Negative
*D. zeae*	A6066	CFBP1889	*A. comosus*	Malaysia	Negative
*D. dadantii*	A5643	CFBP 6467	*Musa* sp.	Martinique	Negative
*D. dadantii*	A6061	CFBP1247	*Dieffenbachia picta*	USA	Negative
*D. dadantii*	A5416	CFBP1269	*Pelargonium capitatum*	Comoro Island (Africa)	Negative
*D. dadantii*	PL193	WI_451_1	*S. tuberosum*	Hawaii, USA	Negative
*D. dadantii*	PL199	WI_249	*S. tuberosum*	Hawaii, USA	Negative
*D. dadantii*	PL200	WI_586	*S. tuberosum*	Hawaii, USA	Negative
*D. paradisiaca*	A5420	CFBP4178	*Musa paradisiaca*	Colombia	Negative
*D. paradisiaca*	A5579	PRI2127	*M. paradisiaca*	Colombia	Negative
*D. dianthicola*	A6059	CFBP3706	*Cichorium intybus*	Switzerland	Negative
*D. dianthicola*	A5572	PRI 1741-B	*S. tuberosum*	Netherlands	Negative
*D. dianthicola*	PL23	GBp10B	*S. tuberosum*	Hawaii, USA	Negative
*D. dianthicola*	PL24	GBp11A	*S. tuberosum*	Hawaii, USA	Negative
*D. dianthicola*	PL25	GBp21C	*S. tuberosum*	Hawaii, USA	Negative
*D. dianthicola*	PL191	WI_367_2	*S. tuberosum*	Hawaii, USA	Negative
*D. dianthicola*	PL192	WI_127_1b	*S. tuberosum*	Hawaii, USA	Negative
*D. dianthicola*	PL194	WI_99_1	*S. tuberosum*	Hawaii, USA	Negative
*D. dianthicola*	PL195	WI_465_2	*S. tuberosum*	Hawaii, USA	Negative
*D. dianthicola*	PL197	WI_47	*S. tuberosum*	Hawaii, USA	Negative
*D. chrysanthemi*	A5641	CFBP 1270	*Parthenium argentatum*	Denmark	Negative
*D. chrysanthemi*	A5415	CFBP2048	*Chrysanthemum morifolium*	USA	Negative
*D. chrysanthemi*	PL196	WI_127_2d	*S. tuberosum*	Hawaii, USA	Negative
*D. chrysanthemi*	PL198	WI_139	*S. tuberosum*	Hawaii, USA	Negative
*D. zeae*	A5422	CFBP2052	*Zea mays*	USA	Negative
*D. zeae*	A5423	CFBP6466	*A. comosus*	Martinique	Negative
*D. zeae*	PL47	F4-3A2	*Brassica oleracea* var. *sabellica*	Hawaii, USA	Negative
*C. michiganensis*	A4775	F293	*S. lycopersicum*	Michigan, USA	Negative
*C. nebraskensis*	A6094	NCPPB2579	*Zea mays*	Nebraska, USA	Negative
*C. sepedonicus*	A2041	R8	*S. tuberosum*	Denmark	Negative
*C. sepedonicus*	A6172	ATCC 33,113	*S. tuberosum*	Canada	Negative
*Rhodococcus fasciens*	A1151	ATCC 12,975	–	USA	Negative
*Curtobacterium flaccumfaciens*	A6266	70,002	*Euphorbia pulcherrima*	–	Negative
*Ralstonia pseudosolanacearum*	A6117	S-6	*Casuarina equisetifolia*	Guam, USA	Negative
*R. solanacearum*	A3450	UW30	*S. lycopersicum*	Trinidad	Negative
*R. syzygii*	A5719	UW521	*Syzygium aromaticum*	–	Negative
*Pantoea agglomerans*	A6222	DP 138	*Z. mays*	Wisconsin, USA	Negative
*Pantoea* sp.	A1869	F7 c. papaya	*Carica papaya*	Hawaii, USA	Negative
*Pantoea* sp.	A5358	J9	*Carica papaya*	Hawaii, USA	Negative
*Xanthomonas phaseoli* pv. *dieffenbachiae*	D182	A6236	*Anthurium andraeanum*	Hawaii, USA	Negative
*X. phaseoli* pv. *dieffenbachiae*	PL37	–	*Anthurium*	Hawaii, USA	Negative
*Bacillus* sp.	A6181	–	–	–	Negative
*Enterobacter asburiae*	A5150	–	*Zingiber officinale*	Hawaii, USA	Negative
*Erwinia amylovora*	A1084	QR-6	*Pyrus* sp.	–	Negative
*Rathayibacter tritici*	LMG 3726	A6287	*Triticum aestivum*	Egypt	Negative
**Endophytes from potato**					
*Pseudomonas* sp.	PL172	S1_WI_465_1	*S. tuberosum*	Hawaii, US	Negative
*Pseudomonas* sp.	PL176	S8_WI_99_2	*S. tuberosum*	Hawaii, US	Negative
*Flavobacterium* sp*.*	PL173	S4_WI_98_1	*S. tuberosum*	Hawaii, US	Negative
*Pantoea* sp.	PL174	S5_WI_451_2	*S. tuberosum*	Hawaii, US	Negative
*Acinetobacter* sp*.*	PL175	S7_WI_451_1	*S. tuberosum*	Hawaii, US	Negative
*Acinetobacter* sp.	PL179	S13_WI_127_1	*S. tuberosum*	Hawaii, US	Negative
*Raoultella* sp.	PL177	S11_WI_367_2	*S. tuberosum*	Hawaii, US	Negative
*Delftia* sp.	PL178	S12_WI_99_2	*S. tuberosum*	Hawaii, US	Negative
**Healthy host**					
*S. tuberosum*					Negative

Negative (−) sign indicates data not available.

### Bacterial colony detection using LAMP assay

**Figure 3:**
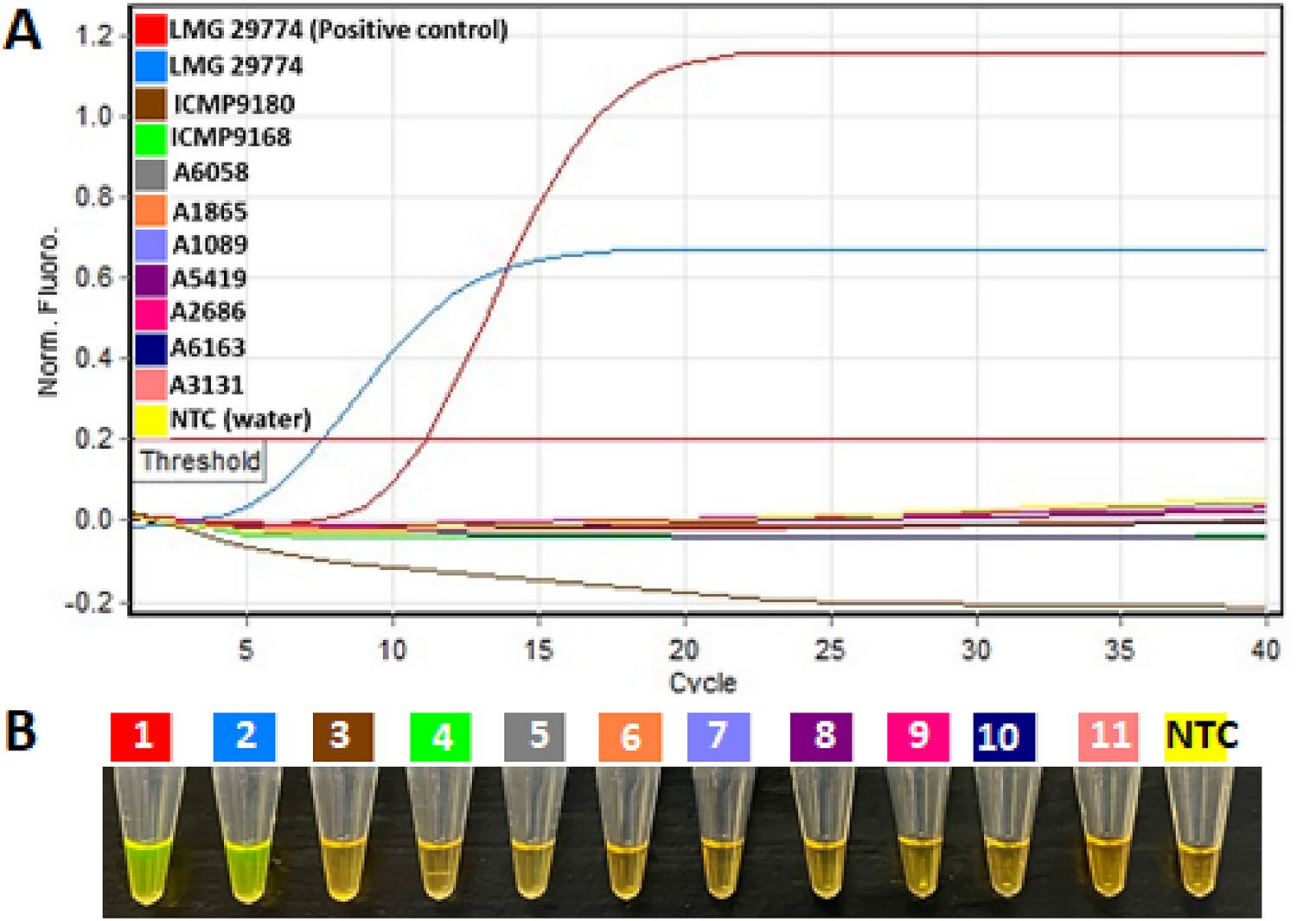
Specific detection of *Pectobacterium parmentieri* using loop-mediated isothermal amplification (LAMP) assay from heat-killed bacterial cells. (**A**) Real-time amplification plot, no sigmoidal curve was observed with strains of exclusivity panel and non-template control; (**B**) visualization of LAMP products after adding 3 μl of SYBR Green I stain. 1- Positive control DNA (*P. parmentieri* LMG29774), 2–11 heat-killed colonies: *P. parmentieri* (LMG29774), *P. polaris* (ICMP 9180), *P. versatile* (ICMP 9168), *D. dianthicola* (A6058), *Pantoea* sp. (A1865), *P. odoriferum* (A1089), *D. dadantii* (A5419), *P. odoriferum* (A2686), *P. atrosepticum* (A6163), *Klebsiella aerogene* (A3131), and negative template control (NTC, water).

The LAMP detection was performed with pure colonies of *P. polaris* (ICMP 9180), *P. versatile* (ICMP 9168), *D. dianthicola* (A6058), *Pantoea* sp. (A1865), *P. odoriferum* (A1089), *D. dadantii* (A 5419), *P. odoriferum* (A2686), *P. atrosepticum* (A6163), *Klebsiella aerogenes* (A3131) and *P. parmentier*i (LMG29774). The DNA template of *P. parmentieri* (LMG29774) and nuclease free water were used as positive and negative controls, respectively. Amplifications were observed with both *P. parmentieri* heat-killed cells and the DNA template (Fig. 3A). Results were validated by adding 3 μL of SYBR Green I (Fig. 3B). No sigmoid curves (Fig. 3A) and subsequent color changes (Fig. 3B) were observed with other non-target strains from exclusivity panel and NTC. Therefore, indicating no cross-reactivity with non-target species. The developed LAMP assay accurately detected the target directly from heat-killed bacterial cells without prior DNA isolation or purification.

### Limit of detection

**Figure 4:**
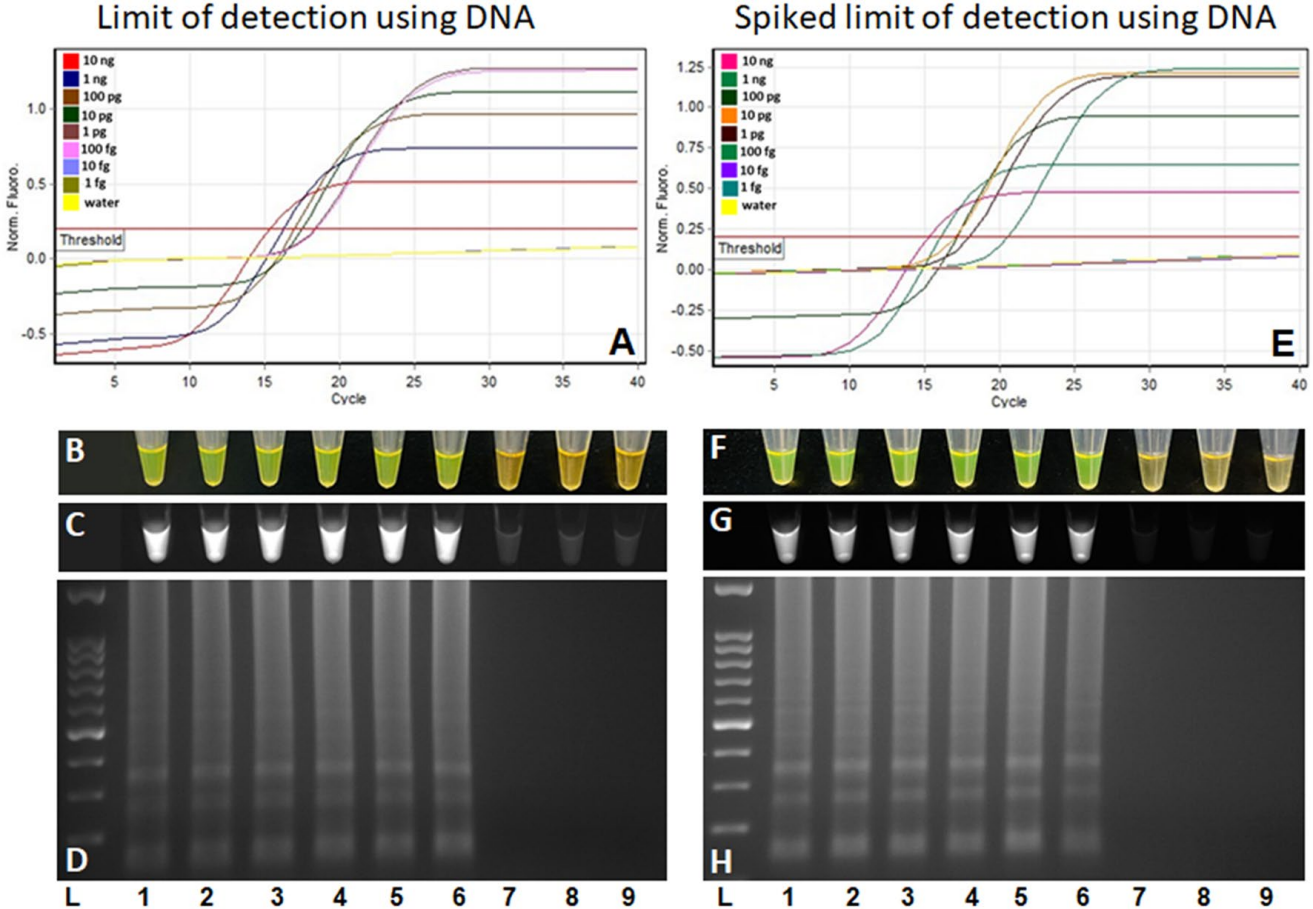
Detection limit of *Pectobacterium parmentieri* specific loop mediated isothermal amplification (LAMP) assay using pure genomic DNA. Ten-fold serially diluted genomic DNA (LMG29774) was used from 10 ng to 1 fg per reaction, indicated with lane 1 to 8. (**A**, **E**) Sigmoidal curves; (**B**, **F**) by adding SYBR Green I dye, color change from orange to bright green observed with naked eyes; (**C**, **G**) tubes observed under the UV light; (**D**, **H**) electrophoresis of amplified products on 2% agarose gel stained with ethidium bromide. Positive amplification was observed up to 100 fg (lane 6). Lane 9 is negative control (non-template control—water) and L is a 100 bp ladder. In the spiked assay, a 5 µl of crude host DNA was added to each reaction containing tenfold serially diluted genomic DNA.

**Figure 5:**
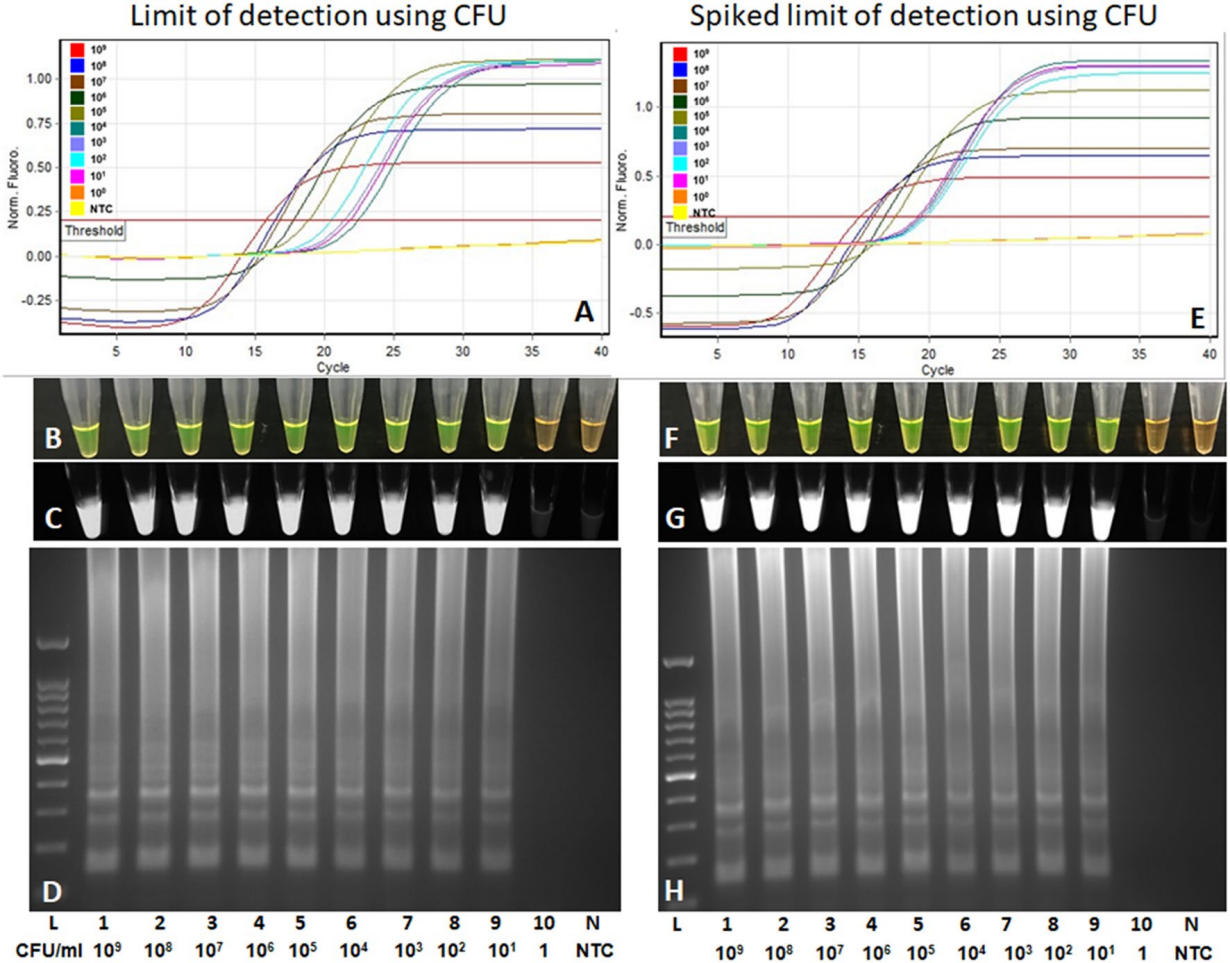
Detection limit of *Pectobacterium parmentieri* specific loop-mediated isothermal amplification (LAMP) assay using heat-killed bacterial cells. Ten-fold serially diluted bacterial cells (LMG 29,774) starting from 10^9^ CFU/mL to 1 CFU/mL per reaction, indicated with lane 1 to 10. (**A**, **E**) Sigmoidal curves; (**B**, **F**) by adding SYBR Green I dye, color change from orange to bright green observed with naked eyes; (**C**, **G**) tubes observed under the UV light; (**D**, **H**) electrophoresis of amplified products on 2% agarose gel stained with ethidium bromide. Positive amplification was observed up to 10 CFU (lane 9). Lane N is negative control (non-template control—water) and L is a 100 bp ladder. In the spiked assay, a 5 µl of crude host DNA was added to each reaction containing tenfold serially diluted heat killed bacterial cells.

The limit of detection was determined using four independent assays with tenfold serially diluted purified genomic DNA and heat-killed bacterial cells. The LAMP assay detected purified *P. parmentieri* genomic DNA down to 100 fg per reaction (Fig. 4A–D). The detection limit with heat-killed cells was 10 CFU/mL (Fig. 5A–D). No adverse effect on the sensitivity was observed when 5 µl of crude host DNA was added in each reaction containing 1 µl of serially diluted genomic DNA (Fig. 4E–H) or lysate of heat-killed cells (Fig. 5E–H). No discrepancies were observed among the results of the different chemistries (fluorescence, SYBR Green I, UV, and gel electrophoresis) used for the cross-validation of the assay (Figs. 4, 5).

### LAMP assay validation with naturally and artificially infected plant and soil samples

The developed assay’s diagnostic capabilities were tested on 14 naturally infected plant samples, 10 artificially inoculated plant samples and 7 artificially infested soil samples. The LAMP assay accurately detected *P. parmentieri* in the DNA isolated from all of the above samples and did not cross-react with samples infected/infested with other *Pectobacterium* or *Dickeya* species (Table 4).

**Table 4:** Validation of *Pectobacterium parmentieri* loop-mediated isothermal amplification (LAMP) with naturally and artificially infected plant and infested soil samples.

Sample ID	Source of DNA	LAMP results	Sample ID	Source of DNA	LAMP results
LMG29774	*Pectobacterium parmentieri*	+	SS9	^b^Naturally infected plant	+
PL70	^a^Potato infected with *P. parmentieri*	+	SS10	^b^Naturally infected plant	+
PL128	^a^Potato infected with *P. parmentieri*	+	SS12	^b^Naturally infected plant	+
PL71	^a^Potato infected with *P. parmentieri*	+	SS23	^b^Naturally infected plant	−
PL72	^a^Potato infected with *P. parmentieri*	+	SS17	^b^Naturally infected plant	−
PL74	^a^Potato infected with *P. parmentieri*	+	SS21	^b^Naturally infected plant	+
PL75	^a^Potato infected with *P. parmentieri*	+	SS19	^b^Naturally infected plant	+
PL67	^a^Potato infected with *P. parmentieri*	+	SS 20	^b^Naturally infected plant	−
PL123	^a^Potato infected with *P. parmentieri*	+	Soil PL128	^c^Soil infested with *P. parmentieri*	+
PL124	^a^Potato infected with *P. parmentieri*	+	Soil PL71	^c^Soil infested with *P. parmentieri*	+
PL73	^a^Potato infected with *P. carotovorum*	−	Soil PL72	^c^Soil infested with *P. parmentieri*	+
SS3	^b^Naturally infected potato	+	Soil PL74	^c^Soil infested with *P. parmentieri*	+
SS1	^b^Naturally infected potato	+	Soil PL75	^c^Soil infested with *P. parmentieri*	+
SS5	^b^Naturally infected potato	+	Soil PL123	^c^Soil infested with *P. parmentieri*	+
SS6	^b^Naturally infected potato	+	Soil PL73	^c^Soil infested with *P. carotovorum*	−
SS7	^b^Naturally infected potato	+	Soil	Negative control	−
SS8	^b^Naturally infected potato	−	Water	Negative template control	−

^a^Potato plants inoculated with *Pectobacterium* sp. in the greenhouse. Samples were taken from a previous study in our lab by Arizala et al.^30^.

^b^Naturally infected potato plant samples were collected in 2019. These samples may have been infected with one or more pectinolytic bacterial species.

^c^Potting soil infested with *Pectobacterium* sp. Samples were taken from a previous study in our lab by Arizala et al.^30^.

‘+’ is positive for *P. parmentieri* and ‘−’ is negative for *P. parmentieri.*

### LAMP validation with artificially inoculated potato tubers to assess the applicability for field applications

**Figure 6:**
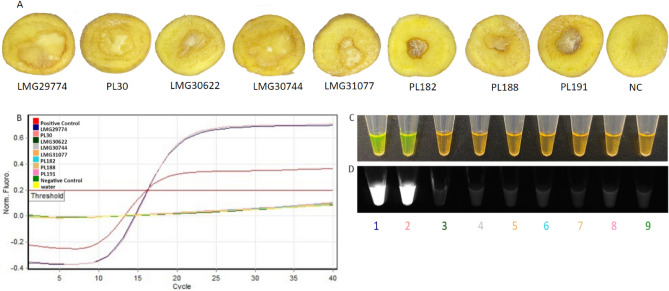
Detection of *Pectobacterium parmentieri* from infected potato samples. (**A**) Infected potato slices infected with different *Pectobacterium* species and *Dickeya dianthicola;* LMG29774 *P. parmentieri*, PL30 *P. parmentieri*, LMG30622 *P. punjabense*, LMG30744 *P. fontis*, LMG31077 *P. polonicum*, PL182 *P. carotovorum*, PL188 *P. wasabiae*, PL191 *D. dianthicola*, NC (negative control) healthy potato. (**B**) Standard curve diagram–only two *P. parmentieri* infected potato slices and positive control LMG29774 *P. parmentieri* were positive, no curve was observed with NC (negative control) healthy potato and NTC, non-template control (water). (**C**) Visualization of LAMP products after addition of SYBR Green I dye—green color represents positive amplification. (**D**) Visualization of SYBR Green I results under UV light—fluorescence indicative of positive amplification. 1, LMG29774 *P. parmentieri;* 2, PL30 *P. parmentieri;* 3, LMG30622 P*. punjabense*; 4, LMG30744 *P. fontis*; 5, LMG31077 *P. polonicum*; 6, PL182 *P. carotovorum*; 7, PL188 *P. wasabiae*; 8, PL191 *D. dianthicola*; 9, NC healthy potato.

Potato slices were inoculated with different *Pectobacterium* species (*P. parmentieri*, *P. punjabense*, *P. fontis*, *P. polonicum*, *P. carotovorum,* and *P. wasabiae*) and *D. dianthocola*. The LAMP assay accurately detected the target pathogen in *P. parmentieri*-inoculated potato tubers. After the addition of SYBR Green, the samples, containing LAMP products, changed from orange to green, indicating a positive amplification. Additionally, no color change was observed for the non-target species, non-template control, or healthy potato slices (Fig. 6).

### Multi-operator validation

Three independent operators performed the LAMP assay with blind samples, including *P. parmentieri* and closely related species, plant material infected with *P. parmentieri*, and a non-template control (Table 5). As indicated, all three operators correctly identified *P. parmentieri* and there was no cross-reactivity with any other non-target samples.

**Table 5:** Multi-operator validation of loop-mediated isothermal (LAMP) assay specific for *Pectobacterium parmentieri*.

Bacteria/infected plant DNA ID	Identity	LAMP test		
		Operator 1	Operator 2	Operator 3
LMG 29,774	*P. parmentieri*	+	+	+
PL74	*P. parmentieri*	+	+	+
Plant infected with PL67	*P. parmentieri*	+	+	+
Plant infected with PL124	*P. parmentieri*	+	+	+
ICMP9180	*P. polaris*	−	−	−
CFBP1357	*P. zantedeschiae*	−	−	−
CFBP8607	*D. fangzhongdai*	−	−	−
Water (NTC)	–	−	−	−

## Discussion

To effectively contain the pathogen and manage the disease, an effective diagnostic test is an essential requirement. Here, we designed and developed a LAMP assay to specifically detect *P. parmentieri* and optimized the test for both field and laboratory diagnostics. LAMP is a popular and well-established rapid and cost-effective diagnostic technique with high specificity and sensitivity which is easily applied at point-of-need. We performed various validation tests to ensure the quality of the assay^31^.

Precision, dependability, and accuracy are important components of a robust and specific detection assay to be utilized in monitoring and surveillance programs. The foundation of a robust and specific assay depends on target selection^32^. The low cost of genome sequencing and availability of whole genomic data in public databases increases the use of comparative genomic approaches for identifying for signature genomic regions exclusively present in target species^32^. In this study, we designed *P. parmentieri* primers to amplify a unique *petF1* gene region, a genomic region highly conserved in all *P. parmentieri* strains tested, but not in other closely related bacterial strains, pathogenic or non-pathogenic (Fig. 1). Comparing the whole genomes of bacteria of different origin and host ranges for target-specific primer development ensures assay’s specificity greatly minimizing inadvertent cross-reactivity with non-target microorganisms and hosts^33^, this virtually eliminating false-positives in the identification of *P. parmentieri*. The designed primers, validated in silico by against the NCBI GenBank database, showed high specificity to *P. parmentieri* (Table 1).

The in-silico validation of primers is required to initially eliminate non-specific targets but does not guarantee the target’s *in-vitro* specificity. Therefore, extensive inclusivity and exclusivity panels were composed of multiple strains of *P. parmentieri* and other closely-related species, respectively (Tables 2, 3). All 15 strains in the inclusivity panel were positive with the LAMP assay (Table 2), while 94 bacterial strains of 18 closely-related species of the genus *Pectobacterium*, including closely-related species that cause similar potato blackleg and soft rot symptoms, were negative (Table 3). Additionally, the assay was tested and validated with endophytic and saprophytic bacteria and DNA from healthy host plants (Table 3). The detection capability and accuracy of an assay can be adversely affected by inclusion of infected plant and soil materials that may contain inhibitors^34^. Similarly, the soil contains compounds that inhibit enzymes involved in DNA manipulation^35^. The developed LAMP assay was not inhibited by naturally or artificially infected plant materials, infested soil samples or plant samples infected with closely-related species. The assay’s accuracy was evaluated by infecting the potato slices with several closely-related species—no false positives or negatives were detected (Fig. 6). The diagnostic assay’s speed and simplicity was achieved by incorporating a Plant Material Lysis Kit (less than 5 min preparation time, as described by Ocenar et al.^22^) with the LAMP assay (10–15 min), which reduced the total assay time to 15–25 min. During validation, neither cross-reactivity nor false positives were observed. Each run included a positive and a negative control.

The high sensitivity of a detection assay reduced the possibilities of false-negative results^15^. Host plant constituents can impact the detection limit of an assay; thus, it is imperative that the detection limits are assessed in the presence of crude plant DNA^22^. The developed LAMP assay’s sensitivity was confirmed by evaluating its performance at low concentrations of genomic DNA and heat-killed bacterial cells. The assay detected purified genomic DNA as low as 100 fg (18–20 genome copies) and a minimum of 10 CFU from bacterial lysate (Fig. 4). The detection limit was not affected by spiking the diluents with host crude plant DNA prepared using Plant Material Lysis Kit (Fig. 5), indicating that our assay will be highly effective in early diagnosis, and identify the pathogen at low concentrations in the plant sap. The dead bacterial cells present in bacterial cell lysate might have contributed to obtain higher sensitivity, and therefore, we believe assessing the assay’s limit of detection using CFU method is not appropriate. The Ocenar and Colleagues^22^ also reported a detection limit of 10 CFU, but reported lower assay sensitivity (1 pg) than was achieved in this study (100 fg) when performed with purified genomic DNA^22^. However, this difference may be due to quantification methods, since NanoDrop quantification is less accurate than the newer Qubit method used in the work reported here. The assay performance can also be affected by the operators, but we have confirmed that the developed assay is repeatable by obtaining concordant results when blind tests were performed by three independent operators (Table 5).

In conclusion, we demonstrated a simplified field-deployable LAMP assay for specific detection of *P. parmentieri*. The assay is sensitive and rapid, and has applications in pathogen detection, quarantine, eradication, border protection, seed certification, disease management, and epidemiology.

## Materials and methods

Any plant and plant materials used in this research compliance with international, national and institutional guidelines.

### Target selection and primer design

A total of 50 complete and draft genomes sequences were included in the analysis; 19 of these genomes came from various *P. parmentieri* strains isolated in different years from distinct geographic locations. All genomes sequences were re-annotated using Prokka^36^. The pan and core genomes among all *Pectobacterium* species were analyzed using the ROARY pipeline^37^. After conducting the pan-core analysis, ROARY output displayed the presence and absence of genes among the 50 genomes, allowing identification of unique gene regions present exclusively in all *P. parmentieri* strains. Candidate genes found exclusively in *P. parmentieri* were analyzed in silico using the nucleotide BLAST algorithm. The *PetF1* gene was identified and used as the specific target for designing the LAMP primers. The criterion for gene selection was to display 100% identity with 100% query coverage of all *P. parmentieri* strains. Additionally, the selected gene had to be absent in the other *Pectobacterium* species and other closely related bacteria that share the same ecological niche of the target pathogen, *P. parmentieri*. After identifying the target gene *petF1*, a nucleotide comparison ring image (Fig. 1) was created to portray the gene’s location and unique presence across different *P. parmentieri* strains. The image was generated using BRIG (BLAST Ring Image Generator)^29^. The genome comparison was performed based upon the NCBI-BLAST version 2.10.0 + database; *P. parmentieri* RNS 08–42-1A served as a reference genome for nucleotide alignment. The complete genomes of six *P. parmentieri* strains and 17 complete/draft genomes other *Pectobacterium* species were included in the analysis. The locus of *petF1* is highlighted in Fig. 1. The genomes included in the circular graphic were downloaded from the NCBI GenBank database with these accession numbers: *P. parmentieri* RNS 08-42-1A (NZ_CP015749), *P. parmentieri* SCC3193 (NC_017845), *P. parmentieri* WPP163 (NC_013421), *P. parmentieri* IFB5619 (NZ_CP026985), *P. parmentieri* HC (NZ_CP046376), *P. parmentieri* IFB5486 (NZ_CP026982), *P*. *actinidiae* KKH3 (NZ_JRMH00000000), *P. aquaticum* A212-S19-A16^T^ (NZ_QHJR00000000), *P. aroidearum* PC1 (NC_012917), *P. atrosepticum* JG10-08 (NZ_CP007744), *P. betavasculorum* NCPPB 2795 (NZ_JQHM00000000), *P. brasiliense* SX309 (NZ_CP020350), *P. carotovorum* WPP14 (NZ_CP051652), *P. fontis* M022^T^ (JSXC00000000), *P. odoriferum* BC S7 (NZ_CP009678), *P. parvum* s0241^T^ (OANP00000000), *P. peruviense* IFB5232^T^ (NZ_LXFV00000000), *P. polaris* NIBIO 1006^T^ (NZ_CP017481), *P. polonicum* DPMP315^T^ (NZ_RJTN00000000), *P. punjabense* SS95^T^ (NZ_CP038498), *P. versatile* 3–2 (NZ_CP024842), *P. wasabiae* CFBP 3304 (NZ_CP015750) and *P. zantedeschiae* 2 M (NZ_PESL00000000).

Six LAMP primers, forward inner primer (Pp-FIP), forward outer primer (Pp-F3), backward inner primer (Pp-BIP), backward outer primer (Pp-B3), forward loop primer (Pp-LF) and backward loop primer (Pp-LB), were designed using PrimerExplorer V5 (https://primerexplorer.jp/e/) and are listed in Table 1. The NCBI GenBank BLASTn tool was used to confirm each primer’s specificity against the available genome database.

### Source of bacterial strains and DNA isolation

A total of 110 bacterial strains from different hosts and geographic locations, including strains obtained from international culture collections, were used in this study (Tables 2, 3). Fifteen strains of *P. parmentieri* and 95 strains belonging to closely-related genera and species were chosen for inclusivity and exclusivity panels including 8 endophytic bacteria, respectively (Tables 2, 3). Bacterial strains listed with “A”, “PL”, and culture collection IDs were stored at − 80 °C, and revived by streaking onto 2,3,5-triphenyltetrazolium chloride (TZC) medium (peptone 10 g 1^−1^, dextrose 5 g l^−1^, 0.001% TZC and agar 17 g l^−1^) and TZC-sucrose medium (TZC-S: peptone 10 g l^−1^, sucrose 5 g l^−1^, 0.001% TZC and agar 17 g l^−1^), respectively (Norman and Alvarez 1989). The plates were incubated at 26 °C (± 2 °C) for 12–24 h. Single colonies were re-streaked onto a new TZC medium plate and later used to harvest pure bacterial growth for DNA isolation^32^.

For bacterial genomic DNA extraction from pure cultures, loopful of bacterial cells from TZC plates was suspended into phosphate‐buffered saline (PBS) or directly into 1.5 mL tubes containing 200 µl alkaline lysis buffer provided and proceed with DNA isolation using the DNeasy Blood and Tissue Kit following the manufacturer’s instruction (Qiagen, Germantown, MD).

### Specificity determination

Specificity of the developed LAMP assay was determined using different bacterial strains included in the inclusivity and exclusivity panels listed in Tables 2 and 3, respectively. The inclusivity panel included 15 strains of *P. parmentieri* isolated from potato (*Solanum tuberosum*) from three different geographical locations (Table 2). Ninety-five samples consisting of all known *Pectobacterium* species, excluding *P. parmentieri*, isolated from different hosts and locations, plant pathogenic Gram-positive and Gram-negative bacteria, potato endophytic bacterial strains and healthy potato plant DNA were included in the exclusivity panel (Table 3). The LAMP reaction of 25 µl consisted of 15 µl Optigene Master Mix (Optigene, West Sussex, UK), 2 µl primer mix (1.6 µM each of Pp-FIP and Pp-BIP, 0.2 µM each of Pp-F3 and Pp-B3, 0.4 µM each of Pp-LF and Dd-LB), 7 µl of water (Invitrogen), and 1 µl DNA template. DNA templates from *P. parmentieri* strains was used as a positive control; DNA from healthy plants and no template DNA (nuclease-free water) were used as the negative control. The LAMP reaction mixture was incubated and amplified in the Rotor-Gene Q (Qiagen, Germantown, MD) at 65 °C for 20 min. The melt curves were analyzed using Rotor-Gene Q series software 2.3.1 (Built 49) at 80–99 °C with an increment of 0.2 °C/s. Positive target amplification was determined by melt curves above a designated threshold. Melt curves below the threshold were deemed as no amplification or negative. The results were also validated using colorimetric-based detection, by adding 3 µl of SYBR Green dye I (Life Technologies Corporation, Eugene, OR) in each amplified reaction. A positive LAMP reaction was indicated by a change in product color from orange to bright green, while negative reactions remained orange. Results obtained using SYBR Green I dye were observed directly either by the naked eye or by placing the reaction tubes under UV light in a Gel Doc XR + Gel Documentation System (Bio-Rad, Hercules, CA).

### LAMP detection directly from heat-killed bacterial colonies

The LAMP detection was performed using colonies of 10 bacterial strains: *P. parmentieri* (LMG29774), *P. polaris* (ICMP 9180), *P. versatile* (ICMP 9168), *D. dianthicola* (A6058), *Pantoea* sp. (A1865), *P. odoriferum* (A1089), *D. dadantii* (A5419), *P. odoriferum* (A2686), *P. atrosepticum* (A6163), and *Klebsiella aerogene* (A3131). Pure DNA template (LMG29774) and nuclease-free water were used as a positive control and non-template control, respectively. Pure colonies from each strain were collected from TZC plates and added to a PCR tube containing 25 μl of nuclease-free water and heated at 95 °C for 10 min in a T-100 thermocycler (Bio-Rad). One microliter of colony lysate was used as a template for LAMP reactions. LAMP assays were performed following the protocol described above. Real-time amplification plots were obtained, and the results validated by adding 3 µl of SYBR Green I dye in the amplified reaction tubes. The tubes were observed directly by the naked eye for color change.

### Limit of detection determination

The detection limit was determined by performing four independent assays—two with genomic DNA and two with heat-killed cells. To determine the limit of detection using genomic DNA, *P. parmentieri* (LMG 29774) purified genomic DNA was quantified using a Qubit 4 fluorometer (Thermo Fisher Scientific, Waltham, MA). Ten-fold serial dilutions were prepared from 10 ng to 1 fg of genomic DNA in nuclease-free water. One microliter of DNA from each dilution was added into the individual LAMP reaction mixture. The LAMP assay was performed following the same conditions and components described above. A spiked assay was performed by adding 5 µl of crude host (potato stem tissues) DNA, prepared using Plant Material Lysis Kit (Optigene, Sussex, UK), in each LAMP reaction while containing 1 µl serially diluted *P. parmentieri* genomic DNA. To determine the limit of detection utilizing heat-killed bacterial cells, an overnight grown culture of *P. parmentieri* (LMG 29774) was tenfold serially diluted and enumerated by spread plating 100 µl of the 10^−6^, 10^−7^ and 10^−8^ cells onto nutrient agar medium (BD, Becton Dickinson) plates in triplicate. Plates were incubated at 28 °C for 18–24 h prior to counting. Bacterial colonies were counted, averaged, and calculated as log_10_ CFU/mL. The count was 1.1 × 10^9^ CFU/mL. For the LAMP assay, bacterial cultures were serially diluted from 10^9^ to 1 CFU in peptone water and heat-killed at 95 °C for 10 min in a T-100 thermocycler, centrifuged at maximum speed for 2 min. One microliter of supernatant from each dilution was used in individual LAMP reactions. LAMP assay conditions and components, except template, were as described above. Spiked assays were performed by adding 5 µl of crude host DNA, as described above, in each LAMP reaction while containing 1 µl of serially diluted heat-killed cells supernatant. A negative control was included in each run.

### Detection from infected plant and soil samples

Naturally and artificially infected plant tissues were used to validate the assay. A total of 14 naturally infected potato plant samples containing *Pectobacterium* and/or *Dickeya* species were screened. A total of 10 infected plants (infected with strains PL67, PL70-75, PL123-124, and PL128) and 7 infected soil samples (infected with strains PL71-75, PL123 and PL128) were included in this study to validate the LAMP protocol. These DNA samples from artificially infected plant and infested soil samples were used from a previous study in our laboratory^30^. These samples were inoculated/infested with known cultures (Table 4) and DNA was isolated using DNeasy PowerSoil Kit (Qiagen) or a DNeasy Plant Mini Kit (Qiagen).

Potato tubers were cleaned using tap water and dipped into a 0.6% hypochlorite solution for 3 min followed by rinsing three times with sterile water then cut into slices. A loopful (~ 10 µl) of overnight grown bacterial culture was inoculated into each potato slice, placed into petri dishes and incubated for 12–18 h. A total of 100 mg macerated tissue was taken and used for crude DNA isolation using a Plant Material Lysis Kit (Optigene, West Sussex, UK). Five μl of crude DNA was used in each LAMP reaction following the above protocol.

### Multi-operator validation

Multi-operator validation was included to confirm the repeatability of the developed assay. Three independent operators performed blind assays with total of 7 samples and one NTC. The samples included genomic DNA from two *P. parmentieri* strains, two DNA samples from *P. parmentieri*-infected plant samples, and DNA from *P. polaris, P. zantedeschiae*, and *D. fangzhongdai* (Table 5). Each operator performed the assay following the LAMP protocol mentioned above. The results were compared with initial diagnostic data.
